# Association between adherence to the Food Guide golden rule and health characteristics among adult Brazilian women: a cross-sectional study with VIGITEL data, 2018-2021

**DOI:** 10.1590/S2237-96222025v34e20240232.en

**Published:** 2025-04-11

**Authors:** Ana Luiza de Souza Almeida, Taciana Maia de Sousa, Thais Cristina Marquezine Caldeira, Rafael Moreira Claro

**Affiliations:** 1 Universidade Federal de Minas Gerais, Escola de Enfermagem, Departamento de Nutrição, Belo Horizonte, MG, Brazil; 2 Universidade do Estado do Rio de Janeiro, Instituto de Nutrição, Departamento de Nutrição Social, Rio de Janeiro, RJ, Brazil; 3 Universidade Federal de Minas Gerais, Faculdade de Medicina, Departamento de Medicina Preventiva e Social, Belo Horizonte, MG, Brazil

**Keywords:** Food Guides, Women’s Health, Diet Healthy, Health Surveys, Noncommunicable Diseases, Guías Alimentarias, Salud de la Mujer, Dieta Saludable, Encuestas Epidemiológicas, Enfermedades crónicas no transmisibles

## Abstract

**Objective:**

To assess association of adherence to the golden rule of the Food Guide for the Brazilian Population with health characteristics among adult women according to sociodemographic characteristics.

**Methods:**

This is a cross-sectional study with 102,057 women interviewed by the Chronic Disease Risk and Protective Factors Surveillance Telephone Survey System in the Brazilian state capital cities and Federal District between 2018 and 2021. Outcome variables included obesity, hypertension, diabetes, depression and negative self-rated health. Adherence to the golden rule was rated by scores (-13 to +12 points) that combined the consumption of ultra-processed foods (negative) and fresh and minimally processed foods (positive). This score was categorized according to consumption tertiles, with low adherence (first tertile), moderate adherence (second tertile) and high adherence (third tertile). Logistic regression was used to calculate the adjusted odds ratios (OR) (by sociodemographic variables) and 95% confidence intervals (95%CI) of the outcomes in relation to adherence to the Guide.

**Results:**

Compared to low adherence, moderate adherence was inversely associated with obesity (OR 0.86; 95%CI 0.78; 0.93) and negative self-rated health (OR 0.72; 95%CI 0.62; 0.84). High adherence was inversely associated with obesity (OR 0.72; 95%CI 0.65; 0.79), hypertension (OR 0.85; 95%CI 0.78; 0.93), depression (OR 0.69; 95%CI 0.59; 0.82) and negative self-rated health (OR 0.55; 95%CI 0.45; 0.67).

**Conclusion:**

Adherence to the Guide’s golden rule was inversely associated with chronic diseases and negative self-rated health among adult Brazilian women.

## Resumen

**Objetivo:**

Avaliar a associação da adesão à regra de ouro do *Guia Alimentar para a População Brasileira* com características de saúde entre mulheres adultas segundo as características sociodemográficas.

**Métodos:**

Trata-se de estudo transversal com 102.057 mulheres entrevistadas pelo Sistema de Vigilância de Fatores de Risco e Proteção para Doenças Crônicas por Inquérito Telefônico nas capitais dos estados e no Distrito Federal entre 2018 e 2021. Variáveis de desfecho incluíram obesidade, hipertensão, diabetes, depressão e autoavaliação negativa de saúde. A adesão à regra de ouro foi obtida por escore (-13 a 12 pontos) que combinou o consumo de alimentos ultraprocessados (negativo) e alimentos in natura e minimamente processados (positivo). Esse escore foi categorizado conforme tercis de consumo, sendo baixa adesão (primeiro tercil), moderada (segundo tercil) e alta adesão (terceiro tercil). Regressão logística foi empregada para calcular a razão de chances (*odds ratio*, OR) ajustada (por variáveis sociodemográficas) e intervalo de confiança de 95% (IC95%) dos desfechos pela adesão ao guia.

**Resultados:**

Comparado à baixa adesão, a adesão moderada foi inversamente associada à obesidade (OR0,86 IC95% 0,78; 0,93) e à autoavaliação negativa de saúde (OR 0,72 IC95% 0,62; 0,84). A alta adesão foi inversamente associada à obesidade (OR 0,72; IC95% 0,65; 0,79), à hipertensão (OR 0,85; IC95% 0,78; 0,93), à depressão (OR 0,69; IC95% 0,59; 0,82) e à autoavaliação negativa de saúde (OR 0,55; IC95% 0,45; 0,67). Conclusão: Adesão à regra de ouro do Guia foi inversamente associada a doenças crônicas e autoavaliação negativa de saúde entre mulheres adultas brasileiras.

## Introduction

The *Food Guide for the Brazilian Population* (1) introduced a new approach to assessing food consumption based on the Nova food classification (2). The central idea is based on the golden rule, which recommends “always prefer natural or minimally processed foods and culinary preparations to ultra-processed foods” (1).

Fresh and minimally processed foods are those not altered by industrial processes that may have undergone minimal processing such as removing inedible or unwanted parts, drying, crushing, grinding, fractionating, roasting, boiling, pasteurization, refrigeration, freezing or packaging (2). These foods are generally prepared with processed culinary ingredients, which are obtained from the fresh food group, such as oils, fats, sugar and salt (2). Processed foods are those produced from the fresh food group with added culinary ingredients to preserve or improve sensory qualities (2). Ultra-processed foods are formulations of ingredients, mostly for exclusively industrial use, that result from various industrial processes (2). Ultra-processed foods are associated with increased risk of obesity (3) and chronic non-communicable diseases, such as diabetes, hypertension and depression (4,5). These foods generally have high energy density, added sugar, sodium, saturated or trans fats, and low amounts of dietary fiber (2).

Despite their harmful effects on health, the consumption of ultra-processed foods increased in Brazil by 5.5% over ten years, from 2008 to 2018 (6). They were estimated to account for 20% of total energy intake, with a slightly higher percentage among women (20.3%) compared to men (19.1%) (7).

In Brazil, women tend to consume less ultra-processed foods (8) in addition to having a lower tendency to accumulate health risk factors, such as smoking, regular consumption of sugary drinks and abusive alcohol consumption (9). Socioeconomic gender disparities negatively impact their ability to access healthier foods, such as fresh and minimally processed foods, due to lower income levels among women (10). This inequality worsens the risk of food insecurity in households headed by women, which are more vulnerable to hunger (11). It is noteworthy that, even with the limitations mentioned, more than half of Brazilian households are headed by women (50.8%) (12). 

Monitoring the food consumption of the female population, as well as its association with health characteristics, becomes extremely relevant for targeting effective public health measures. The objective of this study was to evaluate women’s adherence to the golden rule of the *Food Guide for the Brazilian Population*, according to sociodemographic characteristics and their association with health characteristics in Brazil.

## Methods

### Design

This is a cross-sectional study based on data on 102,057 adult, non-pregnant women. This data was collected by the Chronic Disease Risk and Protective Factors Surveillance Telephone Survey System (*Sistema de Vigilância de Fatores de Risco e Proteção para Doenças Crônicas por Inquérito Telefônico* - VIGITEL) (13).

### Background and participants

VIGITEL is a national survey conducted annually by the Ministry of Health through telephone interviews with adult individuals (≥18 years old) from the 26 Brazilian state capitals and Federal District (13). This study included data collected by the survey between 2018 and 2021, a period in which information on consumption of ultra-processed foods and fresh and minimally processed foods was included in the questionnaire.

The sampling method adopted by VIGITEL consisted of carrying out at least 1,000 interviews per year in each city, allowing a maximum error of three percentage points with a 95% confidence interval (95%CI) (13). For each year, the sampling process began with the random selection of 10,000 fixed-line telephones per city, based on the National Telecommunications Agency registry of residential fixed-line telephones. These lines were organized into replicas with 200 lines that reproduced the same proportion of lines as in the original register. This division was undertaken due to inaccuracy in the *a priori* estimate of the rate of active residential telephone numbers in the register (eligible telephone lines). Once fixed-line eligibility was established, one adult individual from among the residents of each household was selected (simple random sample) and invited to participate in the survey (13).

The VIGITEL estimates were weighted to represent the total adult population of each Federative Unit. The final weight incorporated two factors. One of these corrected the unequal probability of selection (when the household had more than one adult or fixed-line telephone). The second factor adjusted the distribution of the interviewed population (according to age and education) to that projected for the total population in each study, city and year, based on census data and official projections for the population, using the Rake method. Additional information about the methodology used by VIGITEL can be found in the system’s annual reports (13).

### Variables

With effect from 2018, the VIGITEL questionnaire included a block of questions about consumption of 13 subgroups of ultra-processed foods and 12 subgroups of fresh and minimally processed foods. These subgroups were selected based on foods most frequently consumed in Brazil (7).

The questions related to consumption of fresh and minimally processed foods and ultra-processed foods were asked in relation to the previous day, with “yes or no” answer options. To investigate consumption of fresh and minimally processed foods, the following question was asked: “Now I am going to list some foods and I would like you to tell me if you ate any of them yesterday (from when you woke up to when you went to sleep). I’ll start with natural or basic foods: a. Lettuce, kale, broccoli, watercress or spinach; b. Pumpkin, carrot, sweet potato or okra/caruru; c. Papaya, mango, golden melon or pequi; d. Tomato, cucumber, zucchini, eggplant, chayote or beetroot; e. Orange, banana, apple or pineapple; f. Rice, pasta, polenta, couscous or sweetcorn; g. Beans, peas, lentils or chickpeas; h. Potato, cassava or yam; i. Beef, pork, chicken or fish; j. Fried, boiled or scrambled eggs; k. Milk; l. Peanuts, cashew nuts or Brazil nuts.”

To investigate consumption of ultra-processed foods, the following question was asked: “Now I will list processed foods or products: a. Soft drinks; b. Fruit juice in a carton or can; c. Powdered fruit juice; d. Chocolate milk; e. Flavored yogurt; f. Packaged snacks or savory biscuits; g. Sweet biscuits, stuffed biscuits or individual packaged cakes; h. Chocolate, ice cream, jelly, pudding or other industrialized dessert; i. Hotdog sausage, mortadella or ham; j. Bread, hotdog or hamburger bun; k. Mayonnaise, ketchup or mustard; l. Margarine; m. Instant noodles, powdered soup, frozen lasagna or other ready-to-eat frozen meals.”

The score for adherence to the golden rule (always prefer natural or minimally processed foods and culinary preparations to ultra-processed foods) was calculated based on consumption of ultra-processed foods and fresh and minimally processed foods. Consumption of each subgroup of fresh and minimally processed foods added 1 point to the score. Consumption of each ultra-processed food item subtracted 1 point, with the final score varying from -13 to +12 points (Table 1). The greater the consumption of fresh and minimally processed foods and the lower the consumption of ultra-processed foods, the higher the score for adherence to the golden rule. The golden rule adherence score was divided into consumption tertiles, named “low” (first tertile), “moderate” (second tertile) and “high” (third tertile). It is noteworthy that this score consisted of the methodological strategy adopted to combine data on the consumption of ultra-processed, fresh and minimally processed foods into the only indicator that could reflect adherence to the golden rule, instead of evaluating each food group separately.

**Table 1:** Score for each subgroup of ultra-processed and fresh and minimally processed foods used to build the score for adherence to the golden rule of the Food Guide for the Brazilian Population

Ultra-processed food subgroups (negative score)	Score
Soft drinks	-1
Fruit juice in a carton, individual carton or can	-1
Powdered fruit juice	-1
Chocolate drink	-1
Flavored yoghurt	-1
Packaged snacks (or crisps) or salted biscuit	-1
Sweet biscuit, stuffed biscuit or individual packaged cake	-1
Chocolate, ice cream, jelly, flan or other industrialized dessert	-1
Hotdog sausage, sausage, mortadella or ham	-1
Sliced bread, hotdog or hamburger bun	-1
Mayonnaise, ketchup or mustard	-1
Margarine	-1
Instant noodles, packet soup, frozen lasagna or other bought frozen ready meal	-1
Fresh and minimally processed food subgroups (positive score)	Score
Lettuce, kale, broccoli, watercress or spinach	1
Pumpkin, carrot, sweet potato or okra/caruru	1
Papaya, mango, yellow melon or pequi	1
Tomato, cucumber, zucchini, eggplant, chayote or beetroot	1
Orange, banana, apple or pineapple	1
Rice, pasta, polenta, couscous or sweetcorn	1
Beans, peas, lentils or chickpeas	1
Potato, cassava, cará or yam	1
Beef, pork, chicken or fish	1
Fried, boiled or scrambled egg	1
Milk	1
Peanuts, cashew nuts or Brazil nuts	1
Total	-13 to 12

This study investigated women’s health characteristics, such as obesity, negative self-rated health and medical diagnoses of diabetes, hypertension and depression. Obesity was determined based on Body Mass Index (BMI) ≥30 kg/m² (14), calculated from reported weight and height.

The negative self-rated health indicator was obtained through the individual’s classification of their health status as “very good”, “good”, “regular”, “poor” or “very poor”. Participants who answered “poor” or “very poor” were considered to have negative self-assessment of their health.

Prevalence of diabetes, hypertension and depression was determined based on an affirmative answer to the question: “Has a doctor ever told you that you have [name of disease]? (Yes, No, Don’t remember)”. Questions about diabetes and hypertension were available in all years selected for this study. The question about depression was only asked in 2020 and 2021

Sociodemographic information complemented the analyses, including age (18-35, 36-49, 50-64, ≥65 years), schooling (0-8, 9-11, ≥12 years of study), having a partner (yes, no) and race/skin color (Black, mixed race, White, other).

## Statistical methods

The prevalence rates (%) and 95%CIs for sociodemographic information and women’s health characteristics were described according to the level of adherence to the golden rule for the total population and according to sociodemographic groups. Differences between prevalence rates were investigated using the 95%CIs.

Logistic regression models were used to estimate crude and adjusted odds ratios (OR) for associations between women’s adherence to the golden rule and their health characteristics. The adjusted OR were estimated considering sociodemographic information (age, education and marital status) as confounding variables. For this comparison, the reference standard was low adherence to the golden rule.

All analyses were performed using Stata version 16.1, taking a 5% significance level. The VIGITEL system obtained free and informed consent from research participants orally prior to the interview. The VIGITEL databases were available for public use on the official website of the Ministry of Health (http://svs.aids.gov.br/download/Vigitel/).

## Results

We assessed 102,057 adult women. The majority were between 18 and 35 years old (37.09%), had ≥9 years of schooling (37.71%), did not have a partner (56.58%) and were of Black race/skin color (50.31%, of whom 10.80% were Black and 39.51% were of mixed race). The golden rule adherence score ranged from -9 to +12. The highest prevalence of low adherence to the rule was found among women under 35 years old (64.93%), with an intermediate level of education (56.63%), who did not have a partner (57.17%) and were of Black (56.30%) and mixed (53.95%) race/skin color. High adherence to the rule was more prevalent among those over 50 years old (55.19%), with a high level of education (24.20%), with a partner (24.68%) and without significant differences regarding race/ skin color (Table 2).

**Table 2:** Prevalence (%) and confidence intervals (95%CI) of sociodemographic characteristics of Brazilian adult women and adherence to the golden rule of the *Food Guide for the Brazilian Population*. 2018-2021 (n=102,057)

Variables	Population distribution % (95%CI)	Low adherence % (95%CI)	Moderate adherence % (95%CI)	High adherence % (95%CI)
**Age** (years)				
18-35	37.09 (36.23; 37.95)	64.93 (63.41; 66.42)	21.31 (20.07; 22.59)	13.75 (12.77; 14.79)
36-49	25.38 (24.72; 26.03)	50.09 (48.65; 51.54)	27.99 (26.75; 29.26)	21.90 (20.79; 23.05)
50-64	24.67 (24.10; 25.24)	42.78 (41.58; 43.98)	29.35 (28.27; 30.46)	27.86 (26.86; 28.89)
≥65	12.86 (12.55; 13.17)	42.20 (41.15; 43.25)	30.46 (29.50; 31.45)	27.33 (26.44; 28.24)
**Schooling** (years)				
0-8	28.26 (27.58; 28.95)	51.77 (50.39; 53.15)	27.97 (26.80; 29.17)	20.25 (19.28; 21.26)
9-11	37.71 (36.94; 38.46)	56.63 (55.40; 57.86)	24.56 (23.56; 25.59)	18.80 (17.98; 19.65)
≥12	34.03 (33.27; 34.80)	49.34 (47.91; 50.76)	26.45 (25.30; 27.64)	24.20 (23.13; 25.31)
**Has a partner**				
No	56.58 (55.80; 57.35)	57.17 (56.15; 58.18)	24.56 (23.72; 25.42)	18.26 (17.59; 18.97)
Yes	43.42 (42.64; 44.19)	47.05 (45.88; 48.23)	28.26 (27.27; 29.27)	24.68 (23.78; 25.59)
**Race/skin color**				
Black	10.80 (10.24; 11.39)	56.30 (53.54; 59.02)	24.58 (22.49; 26.80)	19.11 (17.17; 21.21)
Mixed race	39.51 (38.75; 40.27)	53.95 (52.74; 55.15)	25.63 (24.63; 26.64)	20.42 (19.55; 21.32)
White	42.16 (41.39; 42.94)	51.21 (50.01; 52.41)	26.90 (25.90; 27.92)	21.89 (21.05; 22.74)
Other	7.52 (7.18; 7.89	50.32 (47.91; 52.73)	27.19 (25.26; 29.21)	22.48 (20.72; 24.35)
Total	-	52.78 (52.00; 53.55)	26.17 (25.53; 26.82)	21.05 (20.49; 21.62)

Among the health characteristics assessed, 21.76% of the women were obese, 27.10% had hypertension, 14.67% had depression and 8.66% had diabetes. Negative self-rated health was reported by 5.71%. No significant differences were found in relation to obesity and depression between the different levels of adherence. The highest prevalence of hypertension (29.52%) and diabetes (10.14%) was found among those with high adherence to the golden rule. Negative self-rated health was more frequent among those with low adherence (6.73%) (Table 3).

**Table 3:** Prevalence (%) and confidence intervals (95%CI) of the health characteristics of Brazilian adult women according to adherence to the golden rule of the *Food Guide for the Brazilian Population*. 2018-2021 (n=102,057)

Health characteristics	Population distribution % (95%CI)	Low adherence % (95%CI)	Moderate adherence % (95%CI)	High adherence % (95%CI)
Obesity	21.76 (21.12; 22.40)	22.91 (21.95; 23.90)	21.66 (20.54; 22.81)	18.99 (17.88; 20.16)
Hypertension	27.10 (26.47; 27.73)	24.94 (24.02; 25.89)	29.52 (28.40; 30.66)	29.49 (28.31; 30.71)
Diabetes	8.66 (8.31; 9.00)	7.53(7.07; 8.02)	10.14 (9.44; 10.88)	9.65 (8.93; 10.42)
Depression	14.67 (13.82; 15.56)	15.39 (14.13; 18.86)	15.14 (13.53; 16.89)	12.28 (10.97; 13.72)
Negative self-rated health	5.71 (5.36; 6.08)	6.73 (6.19; 7.32)	5.15 (4.58; 5.78)	3.84 (3.25; 4.53)

Compared to low adherence to the rule, moderate adherence was inverse to the presence of obesity (adjusted OR 0.86; 95%CI 0.78; 0.93) and negative self-rated health (adjusted OR 0.72; 95% CI 0.62; 0.84). High adherence was inverse to the presence of obesity (adjusted OR 0.72; 95%CI 0.65; 0.79), hypertension (adjusted OR 0.85; 95%CI 0.78; 0.93), depression (adjusted OR 0.69; 95%CI 0.59; 0.82) and negative self-rated health (adjusted OR 0.55; 95%CI 0.45; 0.67) (Table 4).

**Table 4:** Odds ratios (OR) and confidence intervals (95%CI) of health characteristics of Brazilian adult women according to adherence to the golden rule of the *Food Guide for the Brazilian Population*. 2018-2021 (n=102,057)

Characteristic and level of adherence	OR (95%CI)	p-value	Adjusted OR (95%CI)	p-value
Obesity				
Low adherence	1.00		1.00	
Moderate (2^nd^ tertile)	0.92 (0.85; 1.01)	0.101	0.86 (0.78; 0.93)	<0.001
High (3^rd^ tertile)	0.78 (0.71; 0.86)	<0.001	0.72 (0.65; 0.79)	<0.001
Hypertension				
Low (1^st^ tertile)	1.00		1.00	
Moderate (2^nd^ tertile)	1.26 (1.17; 1.35)	<0.001	0.94 (0.86; 1.02)	0.147
High (3^rd^ tertile)	1.25 (1.16; 1.35)	<0.001	0.85 (0.78; 0.93)	0.001
Diabetes				
Low (1^st^ tertile)	1.00		1.00	
Moderate (2^nd^ tertile)	1.38 (1.24; 1.53)	<0.001	1.10 (0.98; 1.23)	0.091
High (3^rd^ tertile)	1.31(1.17; 1.46)	<0.001	0.99 (0.87; 1.12)	0.851
Depression				
Low (1^st^ tertile)	1.00		1.00	
Moderate (2^nd^ tertile)	0.89 (0.70; 1.12)	0.349	0.91 (0.65; 1.04)	0.296
High (3^rd^ tertile)	0.69 (0.55; 0.88)	0.003	0.69 (0.59; 0.82)	<0.001
Negative self-rated health				
Low (1^st^ tertile)	1.00		1.00	
Moderate (2^nd^ tertile)	0.75 (0.64; 0.87)	<0.001	0.72 (0.62; 0.84)	<0.001
High (3^rd^ tertile)	0.55 (0.45; 0.67)	<0.001	0.55 (0.45; 0.67)	<0.001

## Discussion

Based on the analysis of more than 100,000 adult women from 26 Brazilian state capitals and the Federal District, this study investigated association between women’s adherence to the golden rule of the Food Guide for the Brazilian Population and their health characteristics, according to sociodemographic characteristics. The findings indicated that women with low adherence to the rule were younger, with an intermediate level of education, without a partner and of Black and mixed race race/skin color. Those with high adherence to the golden rule were older, with a higher level of education and had a partner.

Compared to women with low adherence to the rule, those with moderate adherence had 14% lower odds of being classified as being obese and 28% lower odds of having negative self-rated health. Women with high adherence had 28% lower odds of being classified as obese, 15% lower odds of having been diagnosed with hypertension, 31% lower odds of having been diagnosed with depression and 45% lower odds of having negative self-rated health.

Global rates of chronic non-communicable diseases are projected to increase 17% over the next decade, with women facing the greatest increase, particularly for obesity, diabetes and depression (15). Although prevalence of hypertension among Brazilian women remained stable between 2006 and 2019 (from 25.4% to 27.2%) (16), prevalence of diabetes increased by 20% in women, from 7.0% to 8.4 %, between 2013 and 2019 (17). Depression was another more prevalent condition among women (6% prevalence compared to 4% among men) (18), being especially relevant among women of reproductive age (13.3%) (19). Obesity is a critical public health issue for women, with projected rates for 2030 of 30.2% (compared to 28.8% for men) in Brazil (20).

The increase in multimorbidity is a worrying trend among Brazilian women. There was a substantial increase in the combination of obesity, diabetes and hypertension, from 5.5% to 9.6% (0.97 percentage points per year), among adult women in the period 2017-2021 (21). This increase was more pronounced in adults who reported negative self-rated health (21). Self-rated health is associated with morbidity and mortality, in which health rated as “poor” or “very poor” is a predictor of a higher risk of mortality (22).

Inadequate eating habits stand out, such as low consumption of fresh and minimally processed foods and high consumption of ultra-processed foods, these being among the most common risk factors for chronic non-communicable diseases (23). Evidence suggests that adhering to dietary guidelines focusing on increasing the consumption of fruit and vegetables, combined with reducing consumption of soft drinks, could prevent 12 million premature deaths annually worldwide. Improving diet quality could reduce premature deaths among women by up to 25% (23). Regular consumption of ultra-processed foods increases the risk of obesity, diabetes, cardiovascular diseases and several other chronic non-communicable diseases (24). Changes in traditional eating patterns have been identified in recent years, such as the reduction in bean consumption, signaling the weakening of Brazil’s traditional food culture. Beans are considered an important marker of healthy eating, included in the fresh and minimally processed food group, but are projected to no longer dominate eating habits by 2025 (25).

Women who deviate from dietary guidelines, such as the golden rule of the Food Guide for the Brazilian Population, may be more susceptible to developing health problems (6,8). The dietary patterns of the female population are considered important in health outcomes of their households, given that women play a fundamental role in planning family meals (10,11). It is imperative to highlight that the guide itself includes guidance on sharing responsibilities for preparing meals among all family members, in order to avoid women being overloaded with domestic activities (1,10).

This situation appears to have deteriorated in recent years, including during the COVID-19 pandemic. Recent trends have shown a 34% drop in grocery purchases made by women and a 146% increase in the use of delivery services (26). There was an increase in consumption of ultra-processed foods, such as cookies, cakes and sweets in 2017-2018 (7). Vegetable consumption decreased from 40.7% to 36.8% among women during the pandemic (27).

The potential adverse health effects of consuming ultra-processed foods (5,4,24) have been publicized, leading to official recommendations to discourage their intake (1). In 2020, food processing was linked to cardiometabolic risk factors and several chronic non-communicable diseases, including obesity, hypertension, diabetes, cardiovascular disease, cancer and depression (5). Encouraging consumption of fresh and minimally processed foods can be as beneficial as avoiding consumption of ultra-processed foods (1,23), as consumption of ultra-processed foods often replaces consumption of fresh and minimally processed foods. This benefits women who adhere to the golden rule in their efforts to combat chronic non-communicable diseases. 

The results of this study emphasized the importance of existing public policies and regulations aimed at promoting healthy eating practices, such as the Promotion of Adequate and Healthy Eating (28). Brazil has implemented programs and policies related to nutrition, food security and socioeconomic factors that influence food consumption. Among them, the Bolsa Família Program (29), which offers financial support to low-income families, and family farming programs (30), which strengthen local food production. It is important to recognize the weaknesses of current regulations, especially with regard to attempts to tax ultra-processed foods, which is under extensive discussion during the ongoing tax reform in Brazil, but little progress has actually been seen (31), as well as regulation of advertising for these products, which still requires significant progress to be made (32).

Some limitations of this study must be considered. The VIGITEL survey was based on self-reported information via telephone interviews, being subject to memory bias or reporting of socially desirable information. Its reach was restricted to individuals with a landline telephone, which could limit the representation of certain population groups. This bias was controlled by applying weighting factors (13), allowing the results obtained to be extrapolated to the total population of the Brazilian state capital cities (13). The validity of the information collected by telephone survey has been investigated in previous studies, which compared VIGITEL data with household surveys (33,34) and found very similar results, demonstrating the reliability of this method when applying sample weights. At a population level, the use of self-reported information has been widely accepted in epidemiological studies, (34-36) being considered a valid parameter for monitoring risk factors for chronic noncommunicable diseases. 

International studies (35,36) corroborate the effectiveness of telephone interviews in large-scale research (28). The absence of validated cutoff points to adequately classify levels of adherence to the Food Guide limited the generalizability of the results of this research. Previous studies sought to analyze the validity and consistency of scales of adherence to the Guide in different subgroups of the population (37) and to understand the influence of sociodemographic factors on adherence to the Guide’s recommendations according to characteristics such as schooling, income and age group (8,37,38), but without focus on clearly presenting cutoff points for analyzing these scores in the population. The need for future research that adequately validates the cutoff points used to categorize adherence to recommended dietary practices stands out. 

This study is relevant when evaluating the golden rule as an independent parameter, while covering both ultra-processed foods and fresh and minimally processed foods together. The use of continuous scales and scores has made it possible to monitor and compare the evolution of health outcomes in the population (9,37,38). In clinical practice or in research to assess food consumption, these tools could be beneficial when used to assess adherence or changes in behavior (37,38). It is believed that the applicability of the score can be of great importance for studies monitoring adherence to the Guide’s recommendations, given that it has a simple metric and is easily compared with other studies.

Moderate and high adherence to the golden rule of the *Food Guide for the Brazilian Population* was inversely associated with obesity, hypertension, depression and negative self-rated health among adult Brazilian women. These findings highlight the relevance of taking the Food Guide recommendations into consideration when promoting women’s health and the importance of public policies aimed at healthy eating habits among this population. We highlight the importance of continuous monitoring of indicators related to the Guide’s impacts on the population’s health. We suggest that future studies be carried out to analyze the temporal trend of adherence to the golden rule and other guidelines contained in the Guide.
