# Supplementary material for: Association between adherence to the Food Guide golden rule and health characteristics among adult Brazilian women: a cross-sectional study with VIGITEL data, 2018-2021
Source: Epidemiol Serv Saude. 2025 Apr 11;34:e20240232. doi: 10.1590/S2237-96222025v34e20240232.en (PMC11998651; doi:10.1590/S2237-96222025v34e20240232.en)
Supplement: Translation

# Translation: Associação entre a adesão à regra de ouro do guia alimentar e as características de saúde entre brasileiras adultas: estudo transversal com dados do Vigitel, 2018-2021

**DOI:** 10.1590/S2237-96222025v34e20240232.pt

**Authors:** Ana Luiza de Souza Almeida, Taciana Maia de Sousa, Thais Cristina Marquezine Caldeira, Rafael Moreira Claro

**Affiliations:** 1 Universidade Federal de Minas Gerais, Escola de Enfermagem, Departamento de Nutrição, Belo Horizonte, MG, Brasil; 2 Universidade do Estado do Rio de Janeiro, Instituto de Nutrição, Departamento de Nutrição Social, Rio de Janeiro, RJ, Brasil; 3 Universidade Federal de Minas Gerais, Faculdade de Medicina, Departamento de Medicina Preventiva e Social, Belo Horizonte, MG, Brasil

**Keywords:** Guias Alimentares, Saúde da Mulher, Dieta Saudável, Inquéritos Epidemiológicos, Doenças Crônicas não Transmissíveis

## Abstract

**Objetivo:**

Avaliar a associação da adesão à regra de ouro do *Guia Alimentar para a População Brasileira* com características de saúde entre mulheres adultas segundo as características sociodemográficas.

**Métodos:**

Trata-se de estudo transversal com 102.057 mulheres entrevistadas pelo Sistema de Vigilância de Fatores de Risco e Proteção para Doenças Crônicas por Inquérito Telefônico nas capitais dos estados e no Distrito Federal entre 2018 e 2021. Variáveis de desfecho incluíram obesidade, hipertensão, diabetes, depressão e autoavaliação negativa de saúde. A adesão à regra de ouro foi obtida por escore (-13 a 12 pontos) que combinou o consumo de alimentos ultraprocessados (negativo) e alimentos in natura e minimamente processados (positivo). Esse escore foi categorizado conforme tercis de consumo, sendo baixa adesão (primeiro tercil), moderada (segundo tercil) e alta adesão (terceiro tercil). Regressão logística foi empregada para calcular a razão de chances (*odds ratio*, OR) ajustada (por variáveis sociodemográficas) e intervalo de confiança de 95% (IC95%) dos desfechos pela adesão ao guia.

**Resultados:**

Comparado à baixa adesão, a adesão moderada foi inversamente associada à obesidade (OR 0,86 IC95% 0,78; 0,93) e à autoavaliação negativa de saúde (OR 0,72; IC95% 0,62; 0,84). A alta adesão foi inversamente associada à obesidade (OR 0,72; IC95% 0,65; 0,79), à hipertensão (OR 0,85; IC95% 0,78; 0,93), à depressão (OR 0,69; IC95% 0,59; 0,82) e à autoavaliação negativa de saúde (OR 0,55; IC95% 0,45; 0,67).

**Conclusão:**

Adesão à regra de ouro do Guia foi inversamente associada a doenças crônicas e autoavaliação negativa de saúde entre mulheres adultas brasileiras.

## Introdução

O *Guia Alimentar para a População Brasileira* (1) introduziu a nova abordagem à avaliação do consumo alimentar com base na classificação Nova de alimentos (2). A ideia central se baseia na regra de ouro, que recomenda “preferir sempre alimentos in natura ou minimamente processados e preparações culinárias a alimentos ultraprocessados” (1).

Os alimentos in natura e minimamente processados são aqueles não alterados por processos industriais que podem ter passado por processamento mínimo como remoção de partes não comestíveis ou indesejadas, secagem, trituração, moagem, fracionamento, torrefação, fervura, pasteurização, refrigeração, congelamento ou envasamento (2). Esses alimentos são geralmente preparados com os ingredientes culinários processados, que são obtidos do grupo de alimentos in natura, como óleos, gorduras, açúcar e sal (2). Os alimentos processados são aqueles produzidos a partir do grupo de alimentos in natura e dos ingredientes culinários para sua preservação ou aprimoramento de qualidades sensoriais (2). Os alimentos ultraprocessados são formulações de ingredientes, em sua maioria de uso exclusivamente industrial, que resultam de vários processos industriais (2). Os alimentos ultraprocessados estão associados ao risco aumentado de obesidade (3) e às doenças crônicas não transmissíveis, como diabetes, hipertensão e depressão (4,5). Esses alimentos geralmente apresentam alta densidade energética, adição de açúcar, sódio, gorduras saturadas ou trans, e baixa quantidade de fibras alimentares (2).

Apesar de seus efeitos nocivos à saúde, o consumo de alimentos ultraprocessados aumentou no Brasil em 5,5% ao longo de dez anos, de 2008 a 2018 (6). Estimou-se que eles constituíam 20% da ingestão total de energia, com percentual ligeiramente maior entre as mulheres (20,3%) em comparação aos homens (19,1%) (7).

No Brasil, as mulheres tendem a consumir menos alimentos ultraprocessados (8) além de menor tendência a acumular fatores de risco à saúde, como tabagismo, consumo regular de bebidas açucaradas e consumo abusivo de álcool (9). As disparidades socioeconômicas de gênero impactam negativamente a sua capacidade de acessar alimentos mais saudáveis, como os in natura e minimante processados, devido aos menores níveis de renda entre as mulheres (10). Essa desigualdade agrava o risco de insegurança alimentar nos lares chefiados por mulheres, que estão mais vulneráveis à fome (11). Ressalta-se que, mesmo com as limitações mencionadas, mais da metade dos lares brasileiros é chefiado por mulheres (50,8%) (12). 

Monitorar o consumo alimentar da população feminina, bem como sua associação com características de saúde, torna-se extremamente relevante para direcionar medidas eficazes de saúde pública. O objetivo deste estudo foi avaliar a adesão das mulheres à regra de ouro do *Guia Alimentar para a População Brasileira*, segundo as características sociodemográficas e sua associação com características de saúde no Brasil.

## Métodos

### Delineamento

Trata-se de estudo transversal com base nos dados de 102.057 mulheres adultas e não gestantes. Esses dados foram coletados pelo Sistema de Vigilância de Fatores de Risco e Proteção para Doenças Crônicas por Inquérito Telefônico (Vigitel) (13). 

### Contexto e participantes

O Vigitel é um inquérito nacional conduzido anualmente pelo Ministério da Saúde por meio de entrevistas telefônicas com indivíduos adultos (≥18 anos) das 26 capitais brasileiras e do Distrito Federal (13). Este estudo incluiu dados coletados pelo inquérito entre 2018 e 2021, período no qual informações sobre o consumo de alimentos ultraprocessados e de alimentos in natura e minimamente processados foram incluídas no questionário.

O método de amostragem adotado pelo Vigitel constituiu-se em realizar pelo menos 1 mil entrevistas por ano em cada cidade, permitindo erro máximo de três pontos percentuais com intervalo de confiança de 95% (IC95%) (13). Para cada ano, o processo de amostragem começou com o sorteio de 10 mil linhas fixas por cidade, a partir do cadastro de linhas residenciais da Agência Nacional de Telecomunicações. Essas linhas foram organizadas em réplicas com 200 linhas que reproduziram a mesma proporção de linhas do cadastro original. Essa divisão foi realizada devido à imprecisão na estimativa a priori da taxa de números residenciais ativos nesses cadastros (linhas elegíveis). Uma vez estabelecida a elegibilidade da linha fixa, um indivíduo adulto entre os residentes de cada domicílio foi selecionado (amostra aleatória simples) e convidado a participar da pesquisa (13).

As estimativas do Vigitel foram ponderadas para representar a população adulta total de cada unidade federativa. O peso final incorporou dois fatores. Um destes corrigiu a probabilidade desigual de seleção (quando o domicílio possuía mais de um adulto ou telefone fixo). O segundo fator ajustou a distribuição da população entrevistada (de acordo com idade e escolaridade) à projetada para a população total em cada estudo, cidade e ano, com base em dados do censo e projeções oficiais para a população, utilizando o método Rake. Informações adicionais sobre a metodologia empregada no Vigitel podem ser encontradas nos relatórios anuais do sistema (13).

### Variáveis

A partir de 2018, o questionário do Vigitel incluiu o segmento de perguntas sobre o consumo de 13 subgrupos de alimentos ultraprocessados e 12 subgrupos de alimentos in natura e minimamente processados. Esses subgrupos foram selecionados com base nos alimentos mais frequentemente consumidos no Brasil (7).

As perguntas relacionadas ao consumo de alimentos in natura e minimamente processados e alimentos ultraprocessados foram feitas em relação ao dia anterior, com opções de respostas “sim ou não”. Para a investigação do consumo de alimentos in natura e minimamente processados, foi aplicada a seguinte pergunta: “Agora vou listar alguns alimentos e gostaria que você me dissesse se comeu algum deles ontem (desde que acordou até quando foi dormir). Vou começar com alimentos naturais ou básicos: a. Alface, couve, brócolis, agrião ou espinafre; b. Abóbora, cenoura, batata-doce ou quiabo/caruru; c. Mamão, manga, melão amarelo ou pequi; d. Tomate, pepino, abobrinha, berinjela, chuchu ou beterraba; e. Laranja, banana, maçã ou abacaxi; f. Arroz, macarrão, polenta, cuscuz ou milho verde; g. Feijão, ervilha, lentilha ou grão-de-bico; h. Batata, mandioca ou inhame; i. Carne bovina, suína, frango ou peixe; j. Ovos fritos, cozidos ou mexidos; k. Leite; l. Amendoim, castanha-de-caju ou castanha-do-Brasil/Pará”. 

Para a investigação do consumo de alimentos ultraprocessados, foi aplicada a seguinte pergunta: “Agora vou listar alimentos ou produtos industrializados: a. Refrigerante; b. Suco de fruta em caixa ou lata; c. Refresco em pó; d. Achocolatado; e. Iogurte com sabor; f. Salgadinhos ou biscoitos salgados embalados; g. Biscoito doce, biscoito recheado ou bolo embalado; h. Chocolate, sorvete, gelatina, pudim ou outra sobremesa industrializada; i. Salsicha, mortadela ou presunto; j. Pão de forma, pão de cachorro-quente ou hambúrguer; k. Maionese, ketchup ou mostarda; l. Margarina; m. Macarrão instantâneo, sopa em pó, lasanha congelada ou outro prato congelado pronto para comer”.

A pontuação de adesão à regra de ouro (preferir sempre alimentos naturais e minimamente processados e preparações culinárias a alimentos ultraprocessados) foi calculada com base no consumo de alimentos ultraprocessados e de alimentos in natura e minimamente processados. O consumo de cada subgrupo de alimentos in natura e minimamente processados adicionou 1 ponto ao escore. O consumo de cada alimentos ultraprocessados subtraiu 1 ponto, com o escore final variando de -13 a 12 pontos (Tabela 1). Quanto maior o consumo de alimentos in natura e minimamente processados e menor o consumo de alimentos ultraprocessados, maior foi a pontuação de adesão à regra de ouro. O escore de adesão à regra de ouro foi dividido em tercis de consumo, denominados como “baixa” (primeiro tercil), “moderada” (segundo tercil) e “alta” (terceiro tercil). Ressalta-se que o referido escore consistiu na estratégia metodológica adotada para combinar os dados de consumo de alimentos ultraprocessados e in natura e minimamente processados no único indicador que pudesse refletir essa adesão à regra de ouro, em vez de avaliar cada grupo alimentar separadamente.

**Table 1:** Pontuação de cada subgrupo de alimento ultraprocessado e in natura e minimamente processado utilizada para construção do escore de adesão à regra de ouro do *Guia Alimentar para a População Brasileira*

Subgrupos de alimentos ultraprocessados (escore negativo)	Pontuação
Refrigerante	-1
Suco de fruta em caixa, caixinha ou lata	-1
Refresco em pó	-1
Bebida achocolatada	-1
Iogurte com sabor	-1
Salgadinho de pacote (ou chips) ou biscoito/bolacha salgado	-1
Biscoito/bolacha doce, biscoito recheado ou bolinho de pacote	-1
Chocolate, sorvete, gelatina, flan ou outra sobremesa industrializada	-1
Salsicha, linguiça, mortadela ou presunto	-1
Pão de forma, de cachorro-quente ou de hambúrguer	-1
Maionese, ketchup ou mostarda	-1
Margarina	-1
Macarrão instantâneo, sopa de pacote, lasanha congelada ou outro prato pronto comprado congelado	-1
Subgrupos de alimentos in natura e minimamente processados (escore positivo)	Pontuação
Alface, couve, brócolis, agrião ou espinafre	1
Abóbora, cenoura, batata-doce ou quiabo/caruru	1
Mamão, manga, melão amarelo ou pequi	1
Tomate, pepino, abobrinha, berinjela, chuchu ou beterraba	1
Laranja, banana, maçã ou abacaxi	1
Arroz, macarrão, polenta, cuscuz ou milho verde	1
Feijão, ervilha, lentilha ou grão de bico	1
Batata comum, mandioca, cará ou inhame	1
Carne de boi, porco, frango ou peixe	1
Ovo frito, cozido ou mexido	1
Leite	1
Amendoim, castanha-de-caju ou castanha-do-Brasil/Pará	1
Total	-13 a 12

Este estudo investigou características de saúde das mulheres, como obesidade, autoavaliação negativa de saúde e diagnósticos médicos de diabetes, hipertensão e depressão. A obesidade foi determinada com base no Índice de Massa Corporal (IMC) ≥30 kg/m² (14), calculado a partir do peso e da altura relatados.

O indicador de autoavaliação negativa de saúde foi obtido por meio da classificação do seu estado de saúde pelo indivíduo como “muito bom”, “bom”, “regular”, “ruim” ou “muito ruim”. Considerou-se a autoavaliação negativa da saúde para aqueles que responderam “ruim” ou “muito ruim”.

A prevalência de diabetes, hipertensão e depressão foi determinada com base na resposta afirmativa à pergunta: “Algum médico já lhe disse que você tem [nome da doença]? (Sim, Não, Não se lembra)”. As questões sobre diabetes e hipertensão estavam disponíveis em todos os anos selecionados para este estudo. A questão sobre depressão foi coletada apenas em 2020 e 2021.

Informações sociodemográficas complementaram as análises, incluindo idade (18-35, 36-49, 50-64, ≥65 anos), escolaridade (0-8, 9-11, ≥12 anos de estudo), presença de companheiro (sim, não) e raça/cor da pele (preta, parda, branca, outros).

### Métodos estatísticos

 As prevalências (%) e IC95% das informações sociodemográficas e as características de saúde das mulheres foram descritas de acordo com o nível de adesão à regra de ouro para a população total e de acordo com os grupos sociodemográficos. As diferenças entre as prevalências foram investigadas pelo IC95%.

Modelos de regressão logística foram usados para estimar as razões de chances (*odds ratio*, OR) brutas e ajustadas para associações entre a adesão das mulheres à regra de ouro e suas características de saúde. A OR ajustada foi estimada considerando as informações sociodemográficas (idade, escolaridade e estado civil) como variáveis de confusão. Para esta comparação, o padrão de referência foi a baixa adesão à regra de ouro.

Todas as análises foram realizadas usando o software Stata versão 16.1, e adotou-se o nível de significância de 5%. O sistema Vigitel obteve o consentimento livre e informado dos participantes da pesquisa oralmente durante a entrevista. Os bancos de dados do Vigitel estavam disponíveis para uso público na página eletrônica oficial do Ministério da Saúde (http://svs.aids.gov.br/download/Vigitel/).

## Resultados

Foram avaliadas 102.057 mulheres adultas. A maioria tinha entre 18 e 35 anos (37,09%), ≥9 anos de escolaridade (37,71%), sem companheiro (56,58%) e de raça/cor da pele negra (50,31%, sendo 10,80% pretos e 39,51% pardos). A pontuação de adesão à regra de ouro variou de -9 a 12. As maiores prevalências de baixa adesão à regra foram observadas entre as mulheres com menos de 35 anos (64,93%), com nível de escolaridade intermediário (56,63%), sem companheiro (57,17%) e raça/cor de pele preta (56,30%) e parda (53,95%). A alta adesão à regra foi mais prevalente entre aquelas com mais de 50 anos (55,19%), com alto nível de escolaridade (24,20%), com companheiro (24,68%) e sem diferenças significativas quanto à raça/cor da pele (Tabela 2).

**Table 2:** Prevalência (%) e intervalos de confiança (IC95%) das características sociodemográficas de mulheres adultas brasileiras e da adesão à regra de ouro do *Guia Alimentar para a População Brasileira*. 2018-2021 (n=102.057)

Variáveis	Distribuição da população % (IC95%)	Adesão baixa % (IC95%)	Adesão moderada % (IC95%)	Adesão alta % (IC95%)
**Idade** (anos)				
18-35	37,09 (36,23; 37,95)	64,93 (63,41; 66,42)	21,31 (20,07; 22,59)	13,75 (12,77; 14,79)
36-49	25,38 (24,72; 26,03)	50,09 (48,65; 51,54)	27,99 (26,75; 29,26)	21,90 (20,79; 23,05)
50-64	24,67 (24,10; 25,24)	42,78 (41,58; 43,98)	29,35 (28,27; 30,46)	27,86 (26,86; 28,89)
≥65	12,86 (12,55; 13,17)	42,20 (41,15; 43,25)	30,46 (29,50; 31,45)	27,33 (26,44; 28,24)
**Escolaridade** (anos)				
0-8	28,26 (27,58; 28,95)	51,77 (50,39; 53,15)	27,97 (26,80; 29,17)	20,25 (19,28; 21,26)
9-11	37,71 (36,94; 38,46)	56,63 (55,40; 57,86)	24,56 (23,56; 25,59)	18,80 (17,98; 19,65)
≥12	34,03 (33,27; 34,80)	49,34 (47,91; 50,76)	26,45 (25,30; 27,64)	24,20 (23,13; 25,31)
**Presença de companheiro**				
Não	56,58 (55,80; 57,35)	57,17 (56,15; 58,18)	24,56 (23,72; 25,42)	18,26 (17,59; 18,97)
Sim	43,42 (42,64; 44,19)	47,05 (45,88; 48,23)	28,26 (27,27; 29,27)	24,68 (23,78; 25,59)
**Raça/cor da pele**				
Preta	10,80 (10,24; 11,39)	56,30 (53,54; 59,02)	24,58 (22,49; 26,80)	19,11 (17,17; 21,21)
Parda	39,51 (38,75; 40,27)	53,95 (52,74; 55,15)	25,63 (24,63; 26,64)	20,42 (19,55; 21,32)
Branca	42,16 (41,39; 42,94)	51,21 (50,01; 52,41)	26,90 (25,90; 27,92)	21,89 (21,05; 22,74)
Outros	7,52 (7,18; 7,89	50,32 (47,91; 52,73)	27,19 (25,26; 29,21)	22,48 (20,72; 24,35)
Total	-	52,78 (52,00; 53,55)	26,17 (25,53; 26,82)	21,05 (20,49; 21,62)

Entre as características de saúde avaliadas, 21,76% das mulheres apresentaram obesidade, 27,10% hipertensão, 14,67% depressão e 8,66% diabetes. A autoavaliação negativa de saúde foi relatada por 5,71%. Não foram observadas diferenças significativas em relação à obesidade e à depressão entre os diferentes níveis de adesão. A maior prevalência de hipertensão (29,52%) e diabetes (10,14%) foi encontrada entre aquelas com alta adesão à regra de ouro. A autoavaliação negativa de saúde foi mais frequente entre aquelas com baixa adesão (6,73%) (Tabela 3). 

**Tabela 3:** Prevalência (%) e intervalos de confiança (IC95%) das características de saúde das mulheres adultas brasileiras segundo adesão à regra de ouro do *Guia Alimentar para a População Brasileira*. 2018-2021 (n=102.057)

Características de saúde	Distribuição da população % (IC95%)	Adesão baixa % (IC95%)	Adesão moderada % (IC95%)	Adesão alta % (IC95%)
Obesidade	21,76 (21,12; 22,40)	22,91 (21,95; 23,90)	21,66 (20,54; 22,81)	18,99 (17,88; 20,16)
Hipertensão	27,10 (26,47; 27,73)	24,94 (24,02; 25,89)	29,52 (28,40; 30,66)	29,49 (28,31; 30,71)
Diabetes	8,66 (8,31; 9,00)	7,53(7,07; 8,02)	10,14 (9,44; 10,88)	9,65 (8,93; 10,42)
Depressão	14,67 (13,82; 15,56)	15,39 (14,13; 18,86)	15,14 (13,53; 16,89)	12,28 (10,97; 13,72)
Autoavaliação negativa de saúde	5,71 (5,36; 6,08)	6,73 (6,19; 7,32)	5,15 (4,58; 5,78)	3,84 (3,25; 4,53)

Comparado à baixa adesão à regra, a adesão moderada foi inversa à presença de obesidade (OR ajustada 0,86; IC95% 0,78; 0,93) e à autoavaliação negativa de saúde (OR ajustada 0,72; IC95% 0,62; 0,84). A alta adesão foi inversa à presença de obesidade (OR ajustada 0,72; IC95% 0,65; 0,79), hipertensão (OR ajustada 0,85; IC95% 0,78; 0,93), depressão (OR ajustada 0,69; IC95% 0,59; 0,82) e autoavaliação negativa de saúde (OR ajustada 0,55; IC95% 0,45; 0,67) (Tabela 4).

**Tabela 4:** Razão de chances (*odds ratio*, OR) e intervalos de confiança (IC95%) de características de saúde de mulheres adultas brasileiras pela adesão à regra de ouro do *Guia Alimentar para a População Brasileira*. 2018-2021 (n=102.057)

Característica e nível de adesão	OR (IC95%)	p-valor	OR ajustada (IC95%)	p-valor
Obesidade				
Adesão baixa	1,00		1,00	
Moderado (2^o^ tercil)	0,92 (0,85; 1,01)	0,101	0,86 (0,78; 0,93)	<0,001
Alto (3^o^ tercil)	0,78 (0,71; 0,86)	<0,001	0,72 (0,65; 0,79)	<0,001
Hipertensão				
Baixo (1^o^ tercil))	1,00		1,00	
Moderado (2^o^ tercil)	1,26 (1,17; 1,35)	<0,001	0,94 (0,86; 1,02)	0,147
Alto (3^o^ tercil)	1,25 (1,16; 1,35)	<0,001	0,85 (0,78; 0,93)	0,001
Diabetes				
Baixo (1^o^ tercil)	1,00		1,00	
Moderado (2^o^ tercil)	1,38 (1,24; 1,53)	<0,001	1,10 (0,98; 1,23)	0,091
Alto (3^o^ tercil)	1,31(1,17; 1,46)	<0,001	0,99 (0,87; 1,12)	0,851
Depressão				
Baixo (1^o^ tercil)	1,00		1,00	
Moderado (2^o^ tercil)	0,89 (0,70; 1,12)	0,349	0,91 (0,65; 1,04)	0,296
Alto (3^o^ tercil)	0,69 (0,55; 0,88)	0,003	0,69 (0,59; 0,82)	<0,001
Autoavaliação negativa de saúde				
Baixo (1^o^ tercil)	1,00		1,00	
Moderado (2^o^ tercil)	0,75 (0,64; 0,87)	<0,001	0,72 (0,62; 0,84)	<0,001
Alto (3^o^ tercil)	0,55 (0,45; 0,67)	<0,001	0,55 (0,45; 0,67)	<0,001

## Discussão

Baseando-se na análise de mais de 100 mil mulheres adultas das 26 capitais brasileiras e do Distrito Federal, este estudo investigou a associação entre a adesão das mulheres à regra de ouro do Guia Alimentar para a População Brasileira e suas características de saúde, segundo as caraterísticas sociodemográficas. Os achados indicaram que mulheres com baixa adesão à regra eram mais jovens, com nível intermediário de escolaridade, sem companheiro e raça/cor de pele preta e parda. Aquelas com alta adesão à regra de ouro eram mais velhas, com nível superior de escolaridade e com companheiro. 

Em comparação com mulheres de baixa adesão à regra, aquelas com adesão moderada tiveram 14% menor chance de serem classificadas com obesidade e 28% menor chance de apresentarem autoavaliação negativa de saúde. As mulheres com alta adesão apresentaram 28% menor chance de serem classificadas com obesidade, 15% menor chance de terem recebido diagnóstico de hipertensão, 31% menor chance de terem recebido diagnóstico de depressão e 45% menor chance de apresentarem autoavaliação negativa de saúde.

As taxas globais de doenças crônicas não transmissíveis foram projetadas para aumentar 17% na próxima década, com as mulheres enfrentando o maior aumento, particularmente para obesidade, diabetes e depressão (15). Embora a prevalência de hipertensão entre as mulheres brasileiras tenha permanecido estável entre 2006 e 2019 (25,4% para 27,2%) (16), a prevalência de diabetes aumentou 20% nas mulheres, de 7,0% para 8,4%, entre 2013 e 2019 (17). A depressão foi outra condição mais prevalente entre mulheres (6% de prevalência em comparação com 4% nos homens) (18), sendo especialmente relevante entre mulheres em idade reprodutiva (13,3%) (19). A obesidade é questão de saúde pública crítica para as mulheres, com taxas projetadas para 2030 de 30,2% (em comparação com 28,8% para os homens) no Brasil (20).

O aumento da multimorbidade é tendência preocupante entre as mulheres brasileiras. Houve aumento substancial na combinação de obesidade, diabetes e hipertensão, de 5,5% para 9,6% (0,97 pontos percentuais por ano), entre mulheres adultas no período 2017-2021 (21). Esse aumento foi mais pronunciado em adultos que relataram autoavaliação negativa de saúde (21). A autoavaliação de saúde associa-se à morbidade e à mortalidade, em que a saúde avaliada como “ruim” ou “muito ruim” é preditora de maior risco de mortalidade (22).

Destacam-se os hábitos alimentares inadequados, como baixo consumo de alimentos in natura e minimamente processados e alto consumo de alimentos ultraprocessados, entre os fatores de risco mais comuns para doenças crônicas não transmissíveis (23). Evidências sugerem que aderir a diretrizes alimentares com foco no aumento do consumo de frutas e vegetais, aliada à redução do consumo de refrigerantes, poderia prevenir 12 milhões de mortes prematuras anualmente no mundo. A melhoria na qualidade da dieta poderia reduzir em até 25% as mortes prematuras em mulheres (23). O consumo regular de alimentos ultraprocessados aumenta o risco de obesidade, diabetes, doenças cardiovasculares e várias outras doenças crônicas não transmissíveis (24). Mudanças nos padrões alimentares tradicionais foram identificadas nos últimos anos, como a redução do consumo de feijão, sinalizando o enfraquecimento da cultura alimentar tradicional do Brasil. O feijão é considerado importante marcador de alimentação saudável incluído no grupo alimentos in natura e minimamente processados, mas está projetado para não mais dominar os hábitos alimentares até 2025 (25).

Mulheres que se afastam das diretrizes alimentares, como a regra de ouro do *Guia Alimentar para a População Brasileira*, podem estar mais suscetíveis a desenvolver problemas de saúde (6,8). Os padrões alimentares da população feminina são considerados importantes nos desfechos de saúde de seus lares, dado que as mulheres desempenham papel fundamental no planejamento das refeições familiares (10,11). É imperativo ressaltar que o próprio guia inclui como orientação o compartilhamento de responsabilidades na preparação de refeições entre todos os membros da família, evitando a sobrecarga das atividades domésticas sobre a mulher (1,10).

Essa situação parece ter se deteriorado nos últimos anos, incluindo durante a pandemia de covid-19. Tendências recentes mostraram queda de 34% nas compras de supermercado feitas por mulheres e aumento de 146% no uso de serviços de entrega (26). Houve aumento no consumo de alimentos ultraprocessados, como biscoitos, bolos e doces em 2017-2018 (7). O consumo de vegetais diminuiu de 40,7% para 36,8% entre as mulheres durante a pandemia (27).

Os potenciais efeitos adversos do consumo de alimentos ultraprocessados na saúde (5,4,24) foram divulgados, levando a recomendações oficiais para desencorajar sua ingestão (1). Em 2020, o processamento de alimentos foi vinculado a fatores de risco cardiometabólicos e várias doenças crônicas não transmissíveis, incluindo obesidade, hipertensão, diabetes, doenças cardiovasculares, câncer e depressão (5). Encorajar o consumo de alimentos in natura e minimamente processados pode ser tão benéfico quanto evitar o consumo de alimentos ultraprocessados (1,23), visto que o consumo de ultraprocessados frequentemente substitui o consumo de alimentos in natura e minimamente processados. Isso beneficia as mulheres que aderem à regra de ouro em seus esforços para combater doenças crônicas não transmissíveis. 

Os resultados deste estudo enfatizaram a importância das políticas públicas e regulamentações existentes voltadas para fomentar práticas alimentares saudáveis, como a Promoção da Alimentação Adequada e Saudável (28). O Brasil implementou programas e políticas relacionadas à nutrição, à segurança alimentar e aos fatores socioeconômicos que influenciam o consumo de alimentos. Entre eles, o Programa Bolsa Família (29), que oferece suporte financeiro a famílias de baixa renda, e os programas de agricultura familiar (30), que fortalecem a produção de alimentos locais. Faz-se importante reconhecer as fragilidades da regulamentação atual, especialmente no que diz respeito às tentativas de taxação de alimentos ultraprocessados, que está em ampla discussão durante a reforma tributária em andamento no país, mas pouco avanço tem sido de fato observado (31), e à regulamentação da publicidade desses produtos, que ainda carecem de avanços significativos (32).

Algumas limitações deste estudo devem ser consideradas. O Vigitel foi baseado em informações autorrelatadas por entrevista telefônica, estando sujeito ao viés de memória ou relato de informações socialmente desejáveis. O alcance foi restrito a indivíduos com telefone fixo, o que poderia limitar a representatividade de certos grupos populacionais. Esse viés foi controlado por meio da aplicação de fatores de ponderação (13), permitindo que os resultados obtidos pudessem ser extrapolados para a população total das capitais brasileiras (13). A validade das informações coletadas por inquérito telefônico foi investigada em estudos prévios, que compararam os dados do Vigitel com inquéritos domiciliares (33,34) e encontrou resultados muito próximos, demonstrando a confiabilidade desse método quando aplicados os pesos amostrais. Em nível populacional, o uso de informações autorrelatadas foi amplamente aceito em estudos epidemiológicos, (34-36) sendo considerado um parâmetro válido para monitoramento de fatores de risco de doenças crônicas não transmissíveis. 

Estudos internacionais (35,36), corroboram a eficácia das entrevistas telefônicas em pesquisas de grande escala (28). A ausência de pontos de corte validados para classificar adequadamente os níveis de adesão ao guia alimentar limitou a capacidade de generalização dos resultados desta pesquisa. Estudos anteriores buscaram analisar a validade e a consistência de escalas de adesão ao guia em diferentes subgrupos da população (37) e a compreensão da influência de fatores sociodemográficos na adesão às recomendações do guia conforme características como escolaridade, renda e faixa etária (8,37,38), mas ainda sem o foco de apresentação clara de pontos de corte para a análise desses escores na população. Destaca-se a necessidade de futuras pesquisas que validem adequadamente os pontos de corte usados para categorizar a adesão às práticas alimentares recomendadas. 

Este estudo é relevante ao avaliar a regra de ouro como parâmetro independente, mas abrangendo tanto alimentos ultraprocessados quanto alimentos in natura e minimamente processados de forma combinada. O uso de escalas contínuas e escores possibilitou o monitoramento e a comparação de evolução de desfechos de saúde na população (9,37,38). Na prática clínica ou em pesquisas para a avaliação do consumo alimentar, essas ferramentas puderam ser benéficas ao serem utilizadas para a avaliação de adesão ou mudanças de comportamentos (37,38). Acredita-se que aplicabilidade do escore pode ser de grande importância para estudos de monitoramento de adesão as recomendações do guia, dado que possui métrica simples, sendo facilmente comparado com outros estudos. 

A adesão moderada e alta à regra de ouro do *Guia Alimentar para a População Brasileira* foi inversamente associada à obesidade, hipertensão, depressão e autoavaliação negativa de saúde entre mulheres adultas brasileiras. Esses achados destacaram a relevância de considerar as recomendações do guia alimentar na promoção da saúde das mulheres e a importância de políticas públicas voltadas para hábitos alimentares saudáveis entre essa população. Ressalta-se a importância do monitoramento contínuo de indicadores relacionados aos impactos do guia na saúde da população. Sugere-se a realização de estudos futuros que analisem a tendência temporal da adesão à regra de ouro e demais diretrizes do guia.
